# Improving the Non-Hydrostatic Numerical Dust Model by Integrating Soil Moisture and Greenness Vegetation Fraction Data with Different Spatiotemporal Resolutions

**DOI:** 10.1371/journal.pone.0165616

**Published:** 2016-12-09

**Authors:** Manzhu Yu, Chaowei Yang

**Affiliations:** NSF Spatiotemporal Innovation Center and Department of Geography and Geoinformation Science, George Mason University, Fairfax, VA, United States of America; Centro de Investigacion Cientifica y de Educacion Superior de Ensenada Division de Fisica Aplicada, MEXICO

## Abstract

Dust storms are devastating natural disasters that cost billions of dollars and many human lives every year. Using the Non-Hydrostatic Mesoscale Dust Model (NMM-dust), this research studies how different spatiotemporal resolutions of two input parameters (soil moisture and greenness vegetation fraction) impact the sensitivity and accuracy of a dust model. Experiments are conducted by simulating dust concentration during July 1–7, 2014, for the target area covering part of Arizona and California (31, 37, -118, -112), with a resolution of ~ 3 km. Using ground-based and satellite observations, this research validates the temporal evolution and spatial distribution of dust storm output from the NMM-dust, and quantifies model error using measurements of four evaluation metrics (mean bias error, root mean square error, correlation coefficient and fractional gross error). Results showed that the default configuration of NMM-dust (with a low spatiotemporal resolution of both input parameters) generates an overestimation of Aerosol Optical Depth (AOD). Although it is able to qualitatively reproduce the temporal trend of the dust event, the default configuration of NMM-dust cannot fully capture its actual spatial distribution. Adjusting the spatiotemporal resolution of soil moisture and vegetation cover datasets showed that the model is sensitive to both parameters. Increasing the spatiotemporal resolution of soil moisture effectively reduces model’s overestimation of AOD, while increasing the spatiotemporal resolution of vegetation cover changes the spatial distribution of reproduced dust storm. The adjustment of both parameters enables NMM-dust to capture the spatial distribution of dust storms, as well as reproducing more accurate dust concentration.

## Introduction

Dust storms have drawn serious concern during the past decades for their impact on the atmospheric environment, biochemical cycles, radiative balance, and human health [1]. Since the late 1980s, several dust models have been developed to simulate and predict the spatiotemporal patterns of the evolution, magnitude, transportation, and deposition of dust storms [2–5]. For example, models with near global coverage, Barcelona Supercomputing Centre-Dust Regional Atmospheric Model 8b v2.0 (BSC-DREAM8b) [6], forecast the atmospheric life cycle of desert dust particles. At the regional level, the Chinese Unified Atmospheric Chemistry Environment for Dust (CUACE/Dust) model [7] is an integral part of a real-time mesoscale sand and dust storm forecasting system for eastern Asia and has an aerosol module differentiating the size of suspended particles. The model used in this study, NMM-dust, has a meteorological core coupled with a dust module [8]. The meteorological core is the non-hydrostatic mesoscale model (NMM), also used in US National Weather Service (NWS) operations. The NMM-dust produces multiple outputs including dust load and concentration up to 3 km spatial resolution.

Although significant progress has been made in the development of sophisticated dust models, results from different models vary substantially despite independent model validations, indicating that the dust models suffer from various degrees of uncertainties [9]. To identify the sources of uncertainties, Todd et al. [10] conducted an inter-comparison of models and concluded that various features account for the uncertainties, including meteorology initialization (e.g., wind speed, direction), land surface initialization (e.g., surface roughness, soil properties), and modeling processes (e.g., emission size distribution, dust optical properties, deposition schemes). It is not possible to partition the uncertainties unless individual aspects are tested through sensitivity experiments, commonly conducted to achieve a better understanding of a single source of uncertainty.

Among the above aspects, initialization of required input parameters plays a significant role by determining the amount of dust emissions from the land surface. Dust emissions are a threshold, sporadic and spatially heterogeneous phenomenon [11] locally controlled on small spatial and temporal scales [12]. Therefore, accurately predicting the magnitude and the spatiotemporal patterns of dust emission remains a crucial challenge for global and regional dust models. Theoretical knowledge currently utilized in models predicts the vertical dust flux if the required input parameters (surface, soil, and meteorological features) are accurately determined [11]. However, the application of complex emission schemes in global, and to a lesser extent regional models, is hampered by the uncertainty of the required input data at the scales of application and the inaccuracies of the meteorological/climate model [13]. This uncertainty is mainly rooted in the fact that dust models require inputs that are typically problematic to quantify, such as soil textural characteristics, soil moisture content, and vegetation characteristics [14–15]. Especially in recent years, the development of high-resolution dust models has created a requirement for high spatiotemporal resolution input data [6], so that the representation of soil and land surface for model initiation needs to be more realistic.

Improved representation of the soil and land surface conditions, especially the parameters that are responsible for dust emission and transport, has the potential of improving model accuracy. Researches have been conducted to test multiple soil and land surface parameters, such as soil properties (texture and moisture), and roughness length. For example, Laurent et al. [11] integrated improved surface features and soil scheme into Regional Atmospheric Modeling System (v6.0). Results demonstrated that the model provides a more realistic simulation of surface winds and improved quantification of regional dust emissions than a previous version. Menut et al. [16] explored the sensitivity of a dust model to the roughness length dataset derived from the European Remote Sensing (ERS) satellite and the soil texture dataset from the State Soil Geographic data base-Food and Agriculture Organization of the United Nations (STATSGO-FAO). They analyzed the model output under the configuration of ERS and STATSGO-FAO, and compared to the Laboratoire Inter-Universitaire des Systemes Atmospheriques (LISA) dataset. Results showed that the configured model output provided more realistic spatial patterns of dust emission than LISA. Similarly, Cheng et al. [17] presented an improved dust emission scheme for application to the global aerosol-climate model ECHAM5-HAM, which updates surface aerodynamic roughness length, soil moisture and East-Asian soil properties. The resulting dust emission output was a more realistic dust distribution verified by available measurements.

However, questions have been raised on that whether the increased resolution will reduce the uncertainty associated with the input data. Valari and Menut [18] investigated the uncertainty of the CHIMERE model by increasing the resolution of input emission data from 48 km to 6 km, and results showed that the model accuracy do not improve monotonously with resolution increases, but that after a certain point discrepancies become larger. Therefore, sensitivity experiments are needed to investigate whether (and to what extent) improving the spatiotemporal resolution of essential inputs (soil moisture and vegetation cover) will impact model accuracy.

The objective of this research is to investigate the sensitivity of the regional dust model, NMM-dust, to two required input data (soil moisture and greenness vegetation fraction) with different spatial and temporal variations. This study seeks to contribute to the literature on the topic in the following ways. First, we select suitable input for the two essential parameters of soil moisture and vegetation cover with different spatial and temporal variations. Second, we examine the impact of the spatiotemporal input variations on NMM-dust result using both ground-based and satellite observations. Third, we provide guidance on the adjustment of the spatiotemporal variations of required input data to improve dust modeling. In the following data and methods section, details are offered on model description, the datasets selected for investigation and observation data for model evaluation, methodology for integrating the proposed model input into model execution, and the evaluation methodology. The experiment and results section provides experimental design, and the evaluation of model results using ground-based and satellite observations for aerosol optical depth. Finally, we conclude and discuss the research, followed by some options of future work.

## Data and Methods

**Fig 1:**
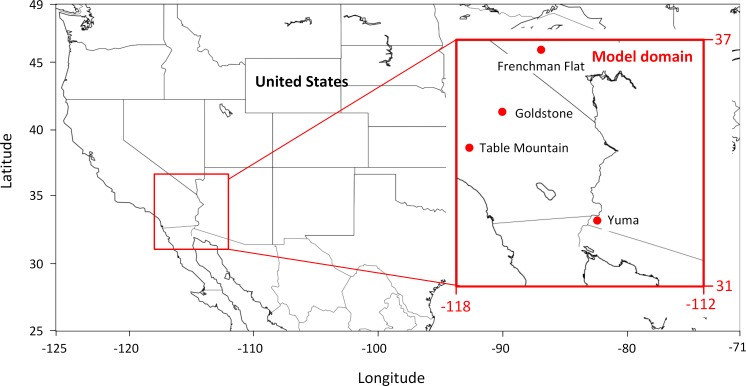
Map of the NMM-dust experiment domain, and four AERONET sites described in Section 4.3.1.

**Fig 2:**
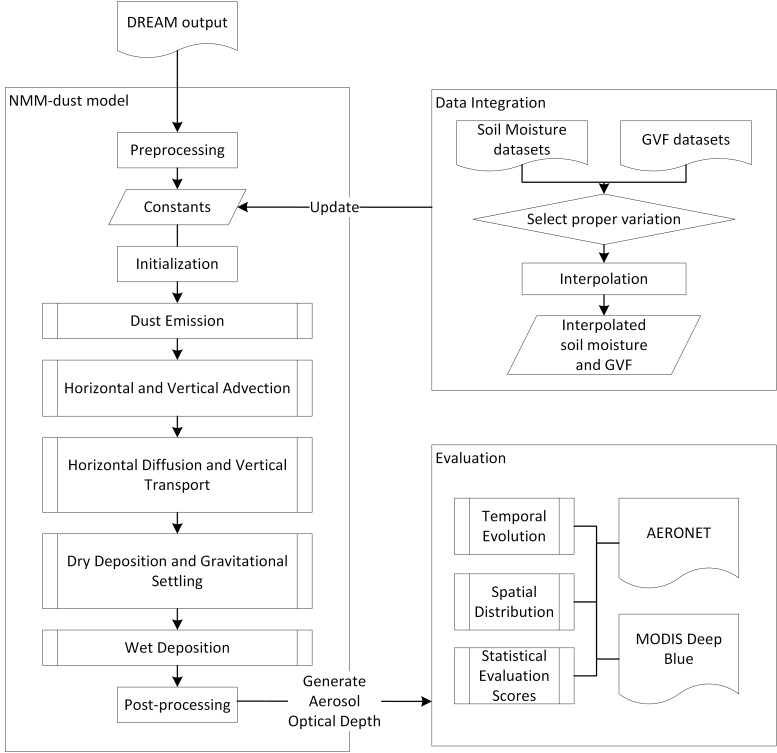
Workflow of NMM-dust, including the integration of soil moisture and GVF datasets, and model evaluation.

In this study, the NMM-dust model is used to compute the dust concentration for the target area covering part of Arizona and California (31, 37, -118, -112), with a resolution of ~ 3 km (Fig 1). The timeframe covers from July 1–7, 2014, during which a dust storm occurred on July 3^rd^ to 4^th^ moving through Phoenix to the south. To test the sensitivity of NMM-dust, we select soil moisture and greenness vegetation fraction dataset with different spatial and temporal resolutions, and integrate them into the initialization stage of NMM-dust, execute experimental model runs, and evaluate experiment results compared with observations. The workflow of input data integration, dust model execution, and result evaluation is schematically illustrated (Fig 2). Herein the model is described, and the geophysical principles of how soil moisture and greenness vegetation fraction affect dust emission are presented in Section 2.1. The description of the proposed model input and observation data used for evaluation is introduced in Section 2.2. The approach of integrating selected input data is introduced in Section 2.3, followed by presentation of evaluation methodology (Section 2.4).

### Model Description

NMM-dust is an atmospheric dust model embedded in the Non-hydrostatic Mesoscale Model [19–20], and is intended to provide around 3–7 days of dust forecasts for regional domains with a horizontal grid spacing to about 3 by 3 km resolution. NMM-dust has been an essential tool that helps improve the understanding of dust emission, transportation, and deposition. The meteorological fields are initialized every 24h (at 0UTC) and boundary conditions are updated every 6h with the NCEP/GFS Global Forecast System Analysis data (1° x 1°). NMM-dust is embedded in the loosely coupled nested models, combining with Dust Regional Atmospheric Model (DREAM) [3, 20]. The loosely coupled nested model was proposed and developed to improve the computing efficiency of dust forecasting. With DREAM producing low resolution dust information over a large domain, NMM-dust can then be executed using DREAM output contributing as initial states to produce high resolution results for sub-regions.

NMM-dust starts with preprocessing, by re-gridding DREAM output (including soil moisture and greenness vegetation fraction) into finer resolution and generates all other required constants into datasets. Initialized with these constants, NMM-dust continues its calculation in the model core, composed of the following processes: 1) dust emission; 2) horizontal and vertical advection; 3) horizontal diffusion and vertical transport; 4) dry deposition and gravitational settling; and 5) wet deposition. NMM-dust includes eight dust size bins with intervals defined by Tegen and Lacis [12] (Table 1).

**Table 1:** Physical and optical properties of dust particle size class.

Size Class	Size Range, *μm*	Effective Radius, *μm*	*Q*_*ext*_(550)	Particle Density, km/m^3^
**1**	0.1–0.18	0.15	1.536	2500
**2**	0.18–0.3	0.25	2.816	2500
**3**	0.3–0.6	0.4	3.086	2500
**4**	0.6–1	0.8	2.583	2500
**5**	1–1.8	1.5	2.3450	2650
**6**	1.8–3	2.5	2.240	2650
**7**	3–6	4	2.174	2650
**8**	6–10	8	2.110	2650

Dust emission is the main process that soil moisture and greenness vegetation fraction affect. Greenness vegetation fraction controls the dust source because dust emission only takes place at the fraction of bare soil exposed in a grid cell, expressed as 1 –GVF. The dust emission scheme allows for soil moisture to affect the uplifting of dust particles. Basically, dust production starts in the form of vertical dust flux for each particle size bin p only when the friction velocity calculated in the model is above the threshold *u*_*ss*_. The threshold friction velocity is calculated as dry soil, which depends on particle size, as Bagnold [21] defined in Eq 1:

$$u_{sd}(p)=A(p)\sqrt{2g\gamma (p)\frac{\rho (p)-\rho_{a}}{\rho_{a}}}$$ (1)

where g is the gravity acceleration, *γ*(*p*) is the particle radius, *ρ*(*p*) is the particle density, and *ρ*_*a*_ is the density of the ambient air. *A*(*p*) is a function of the particle friction Reynolds number for the threshold condition, and is specified using an empirical value [22], indicating that for particles with larger radius the threshold friction velocity is higher. Based on the dry soil threshold, the emission scheme specifies the effect of soil moisture on the threshold friction velocity, as Fécan et al. [23] defined in Eq 2:

$$u_{ss}=u_{sd}\sqrt{1+1.21{\left(w-w\prime \right)}^{0.68}}$$ (2)

where w is the soil moisture content and w’ is the amount of adsorbed water, an increasing function of the clay fraction in the soil.

The output of NMM-dust includes dust concentration, dust load, wind velocity/direction and others. Aerosol optical depth is calculated [12] based on simulated dust load (Eq 3).

$$AOD550=\sum_{1}^{8}AOD550=\sum_{1}^{8}\frac{3}{4\rho \gamma }MQ_{ext}(550)$$ (3)

where *ρ* is the particle mass density, and *γ* is the effective radius. *Q*_*ext*_(550) is the extinction efficiency factor calculated using Mie scattering theory at a wavelength of 550 nm. *M* is the dust mass load.

### Proposed Input and Observation Data

#### Soil moisture data

The development of remote sensing techniques has greatly improved the estimation of soil moisture [24], but these estimates normally bear low temporal resolution, limited surface penetration, and are affected by meteorological conditions and vegetation. In addition, the spatial and vertical structures of soil moisture conditions are nonlinear and depend on quantities not readily observed (i.e., soil texture, precipitation, runoff, infiltration, subsurface drainage) [25]. Another source of soil moisture input commonly used is another model’s output. For example, both BSC-DREAM8b [6] and CHIMERE [26] utilized the National Center for Environmental Prediction (NCEP) data as initial land and meteorological fields. In this approach, the spatial completeness and temporal granularity of the input is assured, but the spatial variation of input soil moisture dataset is not, since the spatial resolutions of two models are normally not consistent. In addition, the accuracy of model output depends on model itself, and needs to be evaluated using land surface and atmospheric observations.

Compared to satellite observations and model data, land data assimilation systems combine the modeled land surface fields with observational estimates, and produce dynamically consistent, spatially complete and temporally continuous estimates of land surface conditions [27]. Land-surface assimilation data has the potential of improving soil moisture analysis [28] and weather prediction [29]. In this sensitivity test, two soil moisture datasets are selected from Land Data Assimilation System (SM_1 and SM_2). The SM_1 [30] is from the Noah land-surface model for Phase 2 of the North American Land Data Assimilation System (NLDAS-2) and has a spatial resolution of 0.125 degree and hourly temporal resolution. Another soil moisture dataset (SM_2) with a different spatiotemporal resolution is from Global Land Data Assimilation System (GLDAS) and has a spatial resolution of 0.25 degree and a temporal resolution of 3 hours. The commonality of these datasets is that the soil layers are uniform across the domain, and both have the same number and thickness of layers as NMM-dust (0–10 cm, 10–40 cm, 40–100 cm, and 100–200 cm underground).

**Fig 3:**
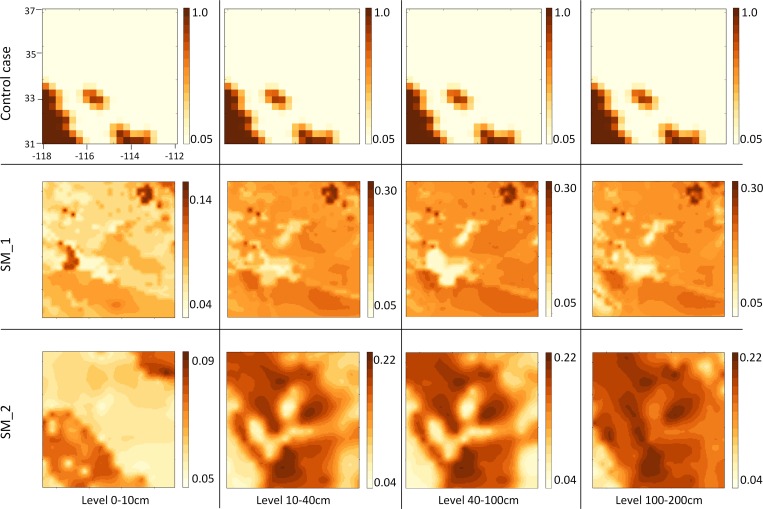
Selected soil moisture datasets at the time of July 1, 2014 00 UTC. Unit in m^3^/m^3^.

The control case (Table 2) is the default model run for NMM-dust; since NMM-dust is coupled with another coarse resolution dust model (DREAM), the inputs to NMM-dust (e.g., soil moisture and vegetation cover) originate from DREAM’s outputs with a spatial resolution of 0.3°. This coarse resolution results in low spatial variation for soil moisture. Specifically, soil moisture values from DREAM output approach 0.05 [*m*^3^/*m*^3^] for all four soil levels within the model domain. Comparing with the soil moisture values from the land data assimilation datasets (Fig 3), the value of 0.05 [*m*^3^/*m*^3^] for all four soil levels does not represent the realistic soil moisture condition, and is smaller than the values from SM_1 and SM_2. Fig 3 shows that soil moisture from the first level (0–10 cm underground) has lower values than those of the other three. Since SM_1 has higher spatial resolution than SM_2, SM_1 and SM_2 show different spatial patterns of soil moisture, but they indicate similar high-valued (northeast) and low-valued (west central) areas. The highest value (dark brown) from SM_1 is greater than that from SM_2 (e.g. Level 1: 0.14 > 0.085).

**Table 2:** Soil moisture datasets.

	DATA SOURCE	RESOLUTION	LEVELS
**CONTROL CASE**	DREAM output	0.3°	4 [0–10, 10–40, 40–100, and 100–200 cm]
**SM_1**	NLDAS-2	Hourly, 0.125°	4 [0–10, 10–40, 40–100, and 100–200 cm]
**SM_2**	GLDAS-1	3-hourly, 0.25°	4 [0–10, 10–40, 40–100, and 100–200 cm]

#### Greenness vegetation fraction data

Vegetation cover is represented in the model as green vegetation fraction (GVF), normally derived from satellite remote sensing and composed into a data product. Since the model starts the emission based on the fraction of bare soil estimated using GVF data, some portions of ground may be covered by plant litter and thus the fraction of bare soil can be overestimated. Therefore, even the study area is mostly covered by open land with dry soil, the model can still overestimate the overall dust emission. When GVF data are available with regional to global coverage, their spatiotemporal resolutions are often too coarse to resolve local scale heterogeneity. For example, the vegetation cover input commonly utilized by dust models is the 5 year-worth of monthly Advanced Very High Resolution Radiometer (AVHRR) data (1985–1990) with 0.144° of spatial resolution, about 15 km in Central America [31]. However, this dataset is outdated and has coarse spatial resolution, which may negatively affect model accuracy and reliability for finer scale simulations. Moreover, the monthly temporal resolution may not provide sufficient information to describe short term variation of vegetation conditions, such as weekly or biweekly [29]. Therefore, GVF input is required with more compatible spatial and temporal resolution for improved model simulations.

Due to the advances in algorithms and techniques of deriving GVF datasets, there are various GVF datasets using different satellite retrievals and methods. For example, Jiang et al. [32] proposed an algorithm to produce real-time weekly GVF data from the Advanced Very High Resolution Radiometer (AVHRR) normalized difference vegetation index (NDVI). This GVF dataset was further investigated using a mesoscale numerical weather prediction model and improved model performance. Broxton et al. [33] developed a 1-km global monthly GVF product for 2001 to 2012 based on Moderate Resolution Imaging Spectroradiometer (MODIS) NDVI and land-cover type. Also derived from MODIS NDVI, daily real-time GVF data [34] are generated by Short-term Prediction Research and Transition (SPoRT) Center with a native 0.01 resolution for the Continental United States (CONUS). To select suitable GVF datasets, it is critical to consider the availability of data for the simulation period and its spatial and temporal variations. Unfortunately, daily real-time GVF from SPoRT is not available for our simulation period. Therefore, weekly and monthly GVF data are used in this research to test the impact on NMM-dust.

In this sensitivity test, two GVF datasets are selected with different spatiotemporal resolutions (GVF_1 and GVF_2). The GVF_1 is derived from the Visible Infrared Imager Radiometer Suite (VIIRS) sensor onboard Suomi National Polar-orbiting Partnership (SNPP) satellite. The GVF_2 is the 5-year (1985 to 1992) monthly mean derived from AVHRR NDVI. Table 3 demonstrates details of the spatiotemporal resolution of datasets utilized in the test.

**Table 3:** Greenness Vegetation Fraction datasets.

	DATA SOURCE	RESOLUTION	MIN-MAX (WITHIN MODEL DOMAIN)
**CONTROL CASE**	DREAM output	0.32°	0.5
**GVF_1**	VIIRS GVF	Weekly, 0.036°	0–0.81
**GVF_2**	5-year AVHRR NDVI (from 1985 to 1992)	Monthly, 0.144°	0–0.96

**Fig 4:**
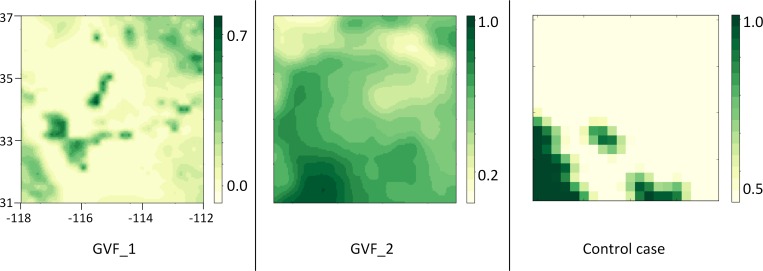
Selected GVF datasets at the time of July 1, 2014 00 UTC. Unit in 1/100.

The datasets for greenness vegetation fraction initializations are illustrated (Fig 4). The GVF’s control case is from DREAM output, and its low spatial variation results in the GVF value within the model domain for the seven days of simulation is ~ 0.5. The comparison of the two GVF datasets interpolated into model resolution at the time of July 1, 2014 00 UTC shows that GVF_1 has more detailed information of vegetation spatial variability than GVF_2, and that the values in GVF_1 are mostly lower than those in GVF_2. In addition, due to different spatial and temporal variations, GVF_1 and GVF_2 show different patterns of vegetation.

#### Observation data

To identify the improvement of dust simulation, model results need to be qualitatively and quantitatively examined. In this research, observation data used for evaluation include the retrieval of column-integrated aerosol properties from Aerosol Robotic Network (AERONET) and aerosol optical depth (AOD) from the MODIS spectrometer travelling on board the NASA Terra and Aqua satellites. AOD information from both observations include other aerosol types, such as sulfate, sea salt, or black carbon. NMM-dust can only generate AOD value transformed from total dust load. To clarify the difference between simulated and observed AODs, we use the report from Arizona Department of Environmental Quality [35] that provides AOD composition information. The aerosol type on July 2nd is majorly dust. The aerosol types on July 3rd include Ozone, PM10 and dust. The aerosol types on July 4th consist of Ozone and dust. Therefore, the observed AOD on July 3rd and 4th should be larger than simulated AOD.

The AERONET [36] is a ground-based remote sensing aerosol network of more than 1000 sites using Sun- and sky-scanning radiometers to measure aerosol optical properties [37]. The AERONET data are used in various satellite and model validation studies as the reference standard for AOD measurements because of its long operational history, sufficient coverage of various regions, and high data quality [38]. Since AERONET sun photometers do not yield AOD at 550 nm (AOD550), this variable is calculated from AOD at 440, 675 and 870 nm (AOD440, AOD675, AOD870) and the Ångström exponent 440–870 (AE440_870) using Ångström law (Eq 4).

$$AOD550=\frac{1}{3}\left(AOD440{\frac{440}{550}}^{AE440\_870}+AOD675{\frac{675}{550}}^{AE440\_870}+AOD870{\frac{870}{550}}^{AE440\_870}\right)$$ (4)

Only closest-in-time observations are considered for model result evaluation. This study uses four sites in the model domain for comparison with model results (Fig 1). These sites are Yuma (32.64° N, 114.58° W, 63.0 m), Frenchman Flat (36.81° N, 115.93° W, 940.0 m), Goldstone (35.23° N, 116.79° W, 1100.0 m), and Table Mountain, CA (34.38° N, 117.68° W, 2200.0 m). The uncertainty of AERONET AOD measurements is reported to be about ±0.01 for AOD at wavelengths greater than 440 nm [36].

As ground observation, AERONET is unavailable under cloudy skies and nighttime conditions and only covers scattered points of an area. Satellite products have the advantage of a large spatial coverage, and the measurements are regular and rapidly available. To complement the point scale observations, MODIS aerosol product is selected with a large spatial coverage at daily intervals. Since the model domain is within a semi-arid area, traditional algorithms do not separate aerosol signals from those of highly reflective surfaces. The DB algorithm [39] takes advantage of the surface phenomenology, performing aerosol retrievals at blue wavelengths (e.g. 0.47 μm spectral channel in MODIS) and utilizing the selected aerosol model in the inversion to generate AOD [39, 40]. In this study, MODIS Level 2 Deep Blue AOD products in 550 nm wavelength are used, with a spatial resolution of 10 × 10 km. Model forecasts are integrated into a 0.11° lat by 0.09° lon grid to match the spatial resolution of MODIS aerosol products. MODIS passed the model domain at ~ 18:00 UTC daily, and with the Deep Blue product, a quality flag was used to filter out unqualified pixels.

### Integrating proposed initialization

To integrate soil moisture and GVF datasets into the model, the constant files generated by preprocessing are modified using the prepared datasets and then used as initialization. The time of these datasets are set according to the time of model’s initiation. For example, if the model is simulating from the time July 1^st^, 2014 00 UTC, the time attribute of the datasets should be as close as possible. Since the target domain configured in the model has a spatial resolution of ~ 3km, integrating selected soil moisture and GVF datasets into the model requires spatial interpolation. Since selected soil moisture datasets have the same soil level settings as NMM-dust, there is no requirement for vertical interpolation. Horizontal interpolation of soil moisture data is conducted using Regression Kriging method [41], using DEM [42], land cover [43], and annual solar radiation as dependent variables. The interpolation of GVF data is conducted using original kriging [44].

### Evaluation methodology

Model results are evaluated using AERONET and MODIS Deep Blue AOD data in 550 nm wavelength (described in Section 3.3). AERONET is used to evaluate the temporal evolution of aerosol condition of a matched location, whereas MODIS Deep Blue AOD data is used to evaluate the spatial distribution of aerosol condition in the covered area. Statistical evaluation scores are calculated for both sources of evaluation. The common metrics that are used to quantify the mean difference between modelled (*c*_*i*_) and observed (*o*_*i*_) quantities are based on the WMO SDS dust forecast model evaluation metrics [45], including the mean bias error (BE), the root mean square error (RMSE), the correlation coefficient (r) and the fractional gross error (FGE).

## Experiments and Results

**Fig 5:**
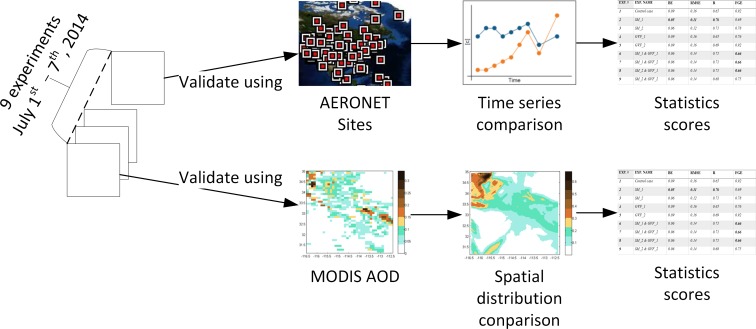
Evaluation procedure.

Nine experiments were conducted regarding different initializations for the simulation period during July 1–7, 2014 (Section 3.1), and the evaluation procedure is illustrated (Fig 5). Two sources of observation data, AERONET and MODIS Deep Blue AOD data, are used to evaluate the nine experimental results. For AERONET data, time series plots are used to compare the temporal evolution of dust with model results for each experiment, and statistic scores are calculated to quantify the model error for each AERONET site. For MODIS Deep Blue AOD data, the spatial distribution of AOD is compared with the model results, along with statistical scores.

### Experiment design

For each day, NMM-dust is executed for nine experiments using different initializations (Table 4). A control run (Experiment 1) is designed using default initializations for both soil moisture and GVF data. Model runs for integrating only one parameter are designed to test the impact of the parameter itself and its spatiotemporal variations in the model (Experiment 2 to 5). Finally, two parameters are both integrated into the model, allowing more variations (Experiment 6 to 9). With this experiment design, the sensitivity of the model to respond to the variation of the soil moisture and GVF condition is tested and the results provide insight for model improvement.

**Table 4:** Experiment design for initialization.

Experiment #	*Initialization*	
	*Soil Moisture*	*GVF*
*1*	Default Initialization	Default Initialization
*2*	Sm_1	Default
*3*	Sm_2	Default
*4*	Default	GVF_1
*5*	Default	GVF_2
*6*	SM_1	GVF_1
*7*	SM_1	GVF_2
*8*	SM_2	GVF_1
*9*	SM_2	GVF_2

### Model result evaluation using AERONET

**Fig 6:**
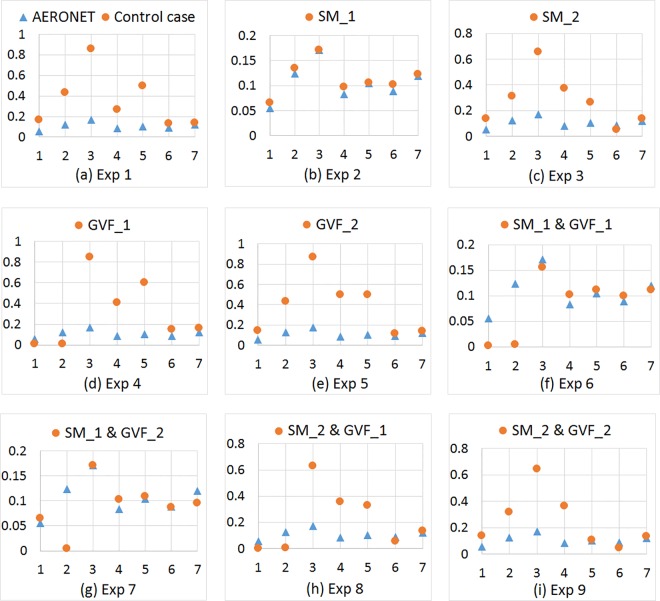
Temporal variations of co-located AERONET observed and NMM-dust simulated aerosol optical depth at 550 nm at site Yuma during July 1–7, 2014. X axis represents time (day), while Y axis represents AOD value for each of nine experiments (a-i).

The time series of NMM-dust simulated aerosol optical depth at 550 nm at four sites in the model domain are compared with co-located AERONET observations during July 1–7, 2014 (Fig 6). Both observation and model results show an increase in AOD on July 3^rd^ and a decrease on July 4^th^ followed by another smaller increase on July 5^th^; this indicates that the simulation properly captures the overall temporal evolution of dust. Taking Site Yuma as an example (Fig 6), the result of control case overestimates the AOD for the entire time period, with the highest value of 0.9 on July 3^rd^, 2014. Adjusting soil moisture in SM_1 effectively reduces the overestimation (0.9 to 0.17), while adjusting in SM_2 also reduces the overestimation to a lesser degree (0.9 to 0.7). On the other hand, adjusting GVF in GVF_1 results in an underestimation for the first two days and does not reduce the overestimation for remainder of the simulation period; while the results of adjusting in GVF_2 is similar to control case result. The AOD values simulated from Experiment 6 and 7 are close to those of Experiment 2 because these three experiments involve the adjustment of soil moisture to SM_1. Similarly, Experiments 8 and 9 similarly reduce the overestimation as in Experiment 3, while results from Experiment 8 show underestimations during the first two days. The comparison of adjusting two (Experiment 6–9) versus one parameter (Experiment 2–5) shows that soil moisture has a stronger impact on the simulated values than GVF.

Besides the time series, evaluation scores using AERONET AOD help explain the patterns and are averaged for the four AERONET sites (Table 5). Positive BE values indicate different degrees of overestimations of the simulated AOD, and the smallest bias occurs when adjusting soil moisture to SM_1. The RMSE represents a similar pattern as that for BE. The correlation coefficients for all experiments range from +0.32 to +0.84, and the best and worst cases are the matches for AERONET with 84% (Exp. 2) and Exp. 4 (32%), respectively. The FGE values indicate that model error ranges from 0.41 to 0.99, indicating that the error increases when adjusting GVF to GVF_1 and decreases when adjusting soil moisture to SM_1. An explanation for this pattern is that SM_1 has higher soil moisture values than the control case and SM_2, which decreases dust emission, whereas GVF_1 has lower vegetation fraction than GVF_2 and the control case, which fails to control dust source of emission.

**Table 5:** Evaluation Scores using AERONET AOD at four Sites (i.e. four data points).

Exp. #	Exp. Name	BE	RMSE	r	FGE
***1***	*Control case*	0.28	0.36	0.36	0.99
***2***	*SM_1*	**0.06**	**0.15**	**0.84**	**0.41**
***3***	*SM_2*	0.16	0.28	0.54	0.97
***4***	*GVF_1*	0.29	0.42	0.32	0.98
***5***	*GVF_2*	0.24	0.39	0.50	0.87
***6***	*SM_1 & GVF_1*	0.08	0.20	0.68	0.51
***7***	*SM_1 & GVF_2*	0.08	0.18	0.66	0.45
***8***	*SM_2 & GVF_1*	0.17	0.28	0.51	0.92
***9***	*SM_2 & GVF_2*	0.17	0.30	0.66	0.55

### Model result evaluation using MODIS Deep Blue data

**Fig 7:**
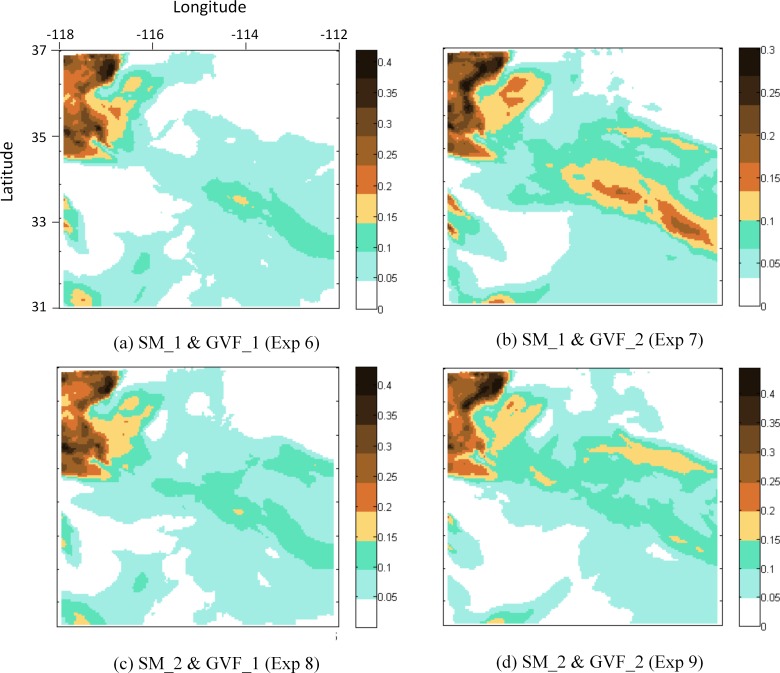
Experiment 6–9 AOD results for July 2^nd^ 2014 18:00 UTC. X axis represents longitude (degree), and Y axis represents latitude (degree). Legends represent AOD values.

**Fig 8:**
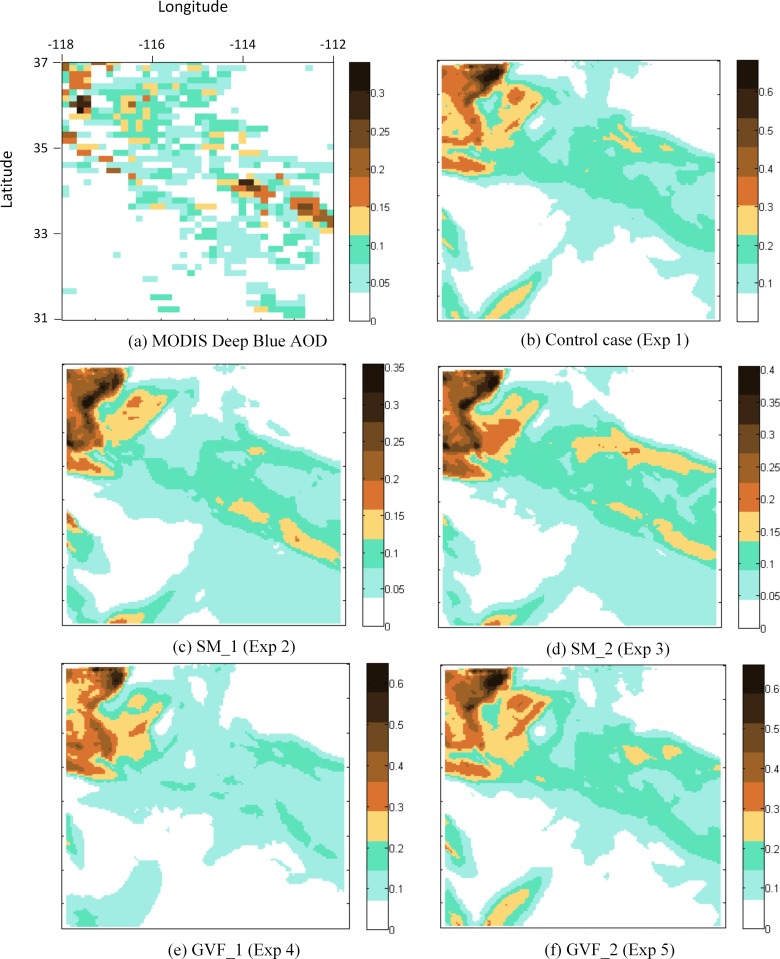
MODIS Deep Blue AOD at July 2^nd^ 2014 18:50 UTC compared with Experiment 1–5 AOD results for July 2^nd^ 2014 18:00 UTC.

MODIS observations of AOD are used to examine the dust storm-induced aerosol changes on a regional scale. The spatial distribution of AOD at 550 nm retrieved by MODIS is compared with the experimental results. Both MODIS Deep Blue and NMM-dust simulated AOD show similar spatial distributions during the simulation period (Fig 5). For example, on July 2^nd^ at ~ 18:00 UTC, the highest values (> 0.3) are found in the northwest of the model domain and less so in the east. Similar to the AERONET evaluation, the control case overestimates MODIS-retrieved AOD. Adjusting soil moisture shows the effective reduction of the overestimation, lowering the highest value from 0.6 (control case) to ~ 0.4 (Exp. 2 and 3), while the spatial coverage of dust remains similar to that of the control. Adjusting the greenness vegetation fraction does not affect the overestimation, with the highest values remaining at 0.6, but changing the spatial coverage of dust. The combination of adjusting soil moisture and GVF constrained the value of AOD (highest values ~ 0.4) but changed the distribution of dust coverage (Fig 7). An example is Experiment 7 (Fig 7B) when adjusting soil moisture to SM_1 and GVF to GVF_2 result in the overestimation of AOD controlled at a value < 0.3. In addition, the locations of high-density areas (Fig 7B), especially two clusters in the east, are associated with those from the MODIS data (Fig 8A), whereas other results (except Fig 8C and 8D, which also involve SM_1) do not reflect this distribution pattern. This result indicates that the high variability of SM_1 increases model accuracy in terms of AOD value, and the combination of SM_1 and GVF_2 better captures the spatial distribution of dust.

Evaluation scores using MODIS Deep Blue data are averaged for seven days (Table 6). Positive BE values demonstrate the overestimation of the model versus MODIS retrieved AOD, and the best value occurs in cases adjusting soil moisture into SM_1 (Exp. 2); the RMSE values show similar patterns. The correlation coefficients indicate that the patterns in the model match ~ 70% of the MODIS Deep Blue, with the greatest match occurring when adjusting SM_1. Furthermore, as a measure of the overall model error, FGE for all experiments range from 0.66 to 0.91, indicating that the control case has the worst accuracy compared with MODIS Deep Blue data, while adjusting soil moisture into SM_1 resulted in the highest accuracy. One notable observation from the evaluation scores is that adjusting only the GVF does not reduce the model error (compare Exp. 4 and 5 with Exp. 1), while adjusting GVF combined with soil moisture (either SM_1 or SM_2) increases model accuracy (Exp. 6–8 with highest FGE scores).

**Table 6:** Evaluation Scores using MODIS Deep Blue data (1833 data points).

EXP. #	EXP. NAME	BE	RMSE	r	FGE
***1***	*Control case*	*0*.*14*	*0*.*25*	*0*.*61*	*0*.*91*
***2***	*SM_1*	***0*.*05***	***0*.*11***	***0*.*76***	*0*.*69*
***3***	*SM_2*	*0*.*09*	*0*.*16*	*0*.*69*	*0*.*83*
***4***	*GVF_1*	*0*.*13*	*0*.*23*	*0*.*56*	*0*.*81*
***5***	*GVF_2*	*0*.*10*	*0*.*19*	*0*.*44*	*0*.*87*
***6***	*SM_1 & GVF_1*	*0*.*06*	*0*.*14*	*0*.*72*	***0*.*66***
***7***	*SM_1 & GVF_2*	*0*.*06*	*0*.*13*	*0*.*70*	*0*.*69*
***8***	*SM_2 & GVF_1*	*0*.*07*	*0*.*15*	*0*.*63*	*0*.*71*
***9***	*SM_2 & GVF_2*	*0*.*07*	*0*.*19*	*0*.*68*	*0*.*75*

## Conclusion and Discussion

This research investigates how spatiotemporal variation of input parameters impacts the sensitivity and accuracy of a dust model (NMM-dust). The selected dust event is the Phoenix dust storm on July 3^rd^, 2014. The time period of July 1^st^ to 7^th^, 2014 was simulated for part of Arizona and California and the spatiotemporal variations of soil moisture and greenness vegetation fraction were tested. Two soil moisture datasets with different spatiotemporal resolution were selected from land data assimilation systems, while two greenness vegetation fraction datasets were selected from satellite derived composite data. These datasets were integrated into NMM-dust initialization separately or in combination to examine the impact of their spatiotemporal variations. Evaluations were conducted using ground (AERONET) and satellite (MODIS) based observations. Results from the AERONET evaluation indicated that although NMM-dust result overestimates the observation, the model captured the temporal evolution of dust storm. In addition, adjusting soil moisture to a higher spatiotemporal resolution effectively reduced the model’s overestimation. The spatiotemporal distribution of simulated dust coverage is validated using MODIS Deep Blue data. Adjusting soil moisture reduced the overestimation of dust volume, while adjusting greenness vegetation fraction changed the spatial distribution of dust storm. Evaluation scores demonstrated that the higher spatiotemporal resolution of soil moisture parameter affected the model’s accuracy, while the combination of adjusting soil moisture and GVF captured the spatial distribution of dust concentration. However, adjusting only GVF did not affect the model’s accuracy. Overall, the result indicated that NMM-dust is able to qualitatively reproduce the observed variations in AOD, and adjusting input parameters enables NMM-dust to perform the reproduction quantitatively.

There might be various reasons why model performance is more sensitive to soil moisture resolution than to vegetation cover, and we raise two of them as hypotheses. Firstly, this sensitivity test is based on soil moisture and vegetation cover data that are available, hence we cannot find GVF dataset with higher spatiotemporal resolution, such as daily or hourly. The GVF dataset used in this study is a composite of multiple days or years, and they are assumed to be valid at the mid-point (GVF_1: fourth day of each week, GVF_2: fifteenth day of each month) [31]. The valid values of both datasets are not completely consistent with the simulation period, which is the first week of July. This inconsistency of valid values for GVF datasets might contribute to the result that the model accuracy is less affected by GVF. Secondly, vegetation cover is not the only vegetation-related impacting factor in surface control of dust emission [46]. The dust emission scheme also takes account of vegetation type and their changes. Therefore, adjusting GVF without changing vegetation type might have less impact on model performance in controlling surface dust emission.

Spatiotemporal variations of input data have a significant impact on model performance, and appropriate selection of the variation combinations improves model accuracy. Future research will investigate additional input variables, including vegetation type, topology, and soil temperature. Model sensitivities on spatiotemporal variations of these variables will be tested using the methodology described herein. It is proposed as a significantly useful scheme of input data adjustment for improving dust storm modeling. The introduction of more variables into the modeling process will also require study on better scheduling [47], big data processing [48] and spatiotemporal computing techniques [49, 50] to satisfy forecasting needs.
